# Pathways for best practice diffusion: the structure of informal relationships in Canada’s long-term care sector

**DOI:** 10.1186/s13012-017-0542-7

**Published:** 2017-02-03

**Authors:** James W. Dearing, Amanda M. Beacom, Stephanie A. Chamberlain, Jingbo Meng, Whitney B. Berta, Janice M. Keefe, Janet E. Squires, Malcolm B. Doupe, Deanne Taylor, Robert Colin Reid, Heather Cook, Greta G. Cummings, Jennifer L. Baumbusch, Jennifer Knopp-Sihota, Peter G. Norton, Carole A. Estabrooks

**Affiliations:** 1 Department of Communication, Michigan State University, Suite 473, 404 Wilson Road, East Lansing, MI 48824-1212 USA; 2 Faculty of Nursing, University of Alberta, Edmonton, Alberta Canada; 3 Dalla Lana School of Public Health, University of Toronto, Toronto, Ontario Canada; 4 Nova Scotia Centre on Aging, Mount Saint Vincent University, Halifax, Nova Scotia Canada; 5 School of Nursing, University of Ottawa, Ottawa, Ontario Canada; 6 Manitoba Center for Health Policy, University of Manitoba, Winnipeg, Canada; 7 Research Department, Interior Health Authority, Kelowna, British Columbia Canada; 8 School of Health and Exercise Sciences, University of British Columbia Okanagan, Kelowna, British Columbia Canada; 9 Hospitals and Communities Integrated Services, Interior Health Authority, Kelowna, British Columbia Canada; 10 School of Nursing, University of British Columbia, Vancouver, British Columbia Canada; 11 Faculty of Health Disciplines, Athabasca University, Athabasca, Alberta Canada; 12 Department of Family Medicine, University of Calgary, Calgary, Alberta Canada

**Keywords:** Canada, Long-term care sector, Long-term care, Diffusion of innovations, Advice seeking, Social network analysis, Opinion leadership, Integrated knowledge translation

## Abstract

**Background:**

Initiatives to accelerate the adoption and implementation of evidence-based practices benefit from an association with influential individuals and organizations. When opinion leaders advocate or adopt a best practice, others adopt too, resulting in diffusion. We sought to identify existing influence throughout Canada’s long-term care sector and the extent to which informal advice-seeking relationships tie the sector together as a network.

**Methods:**

We conducted a sociometric survey of senior leaders in 958 long-term care facilities operating in 11 of Canada’s 13 provinces and territories. We used an integrated knowledge translation approach to involve knowledge users in planning and administering the survey and in analyzing and interpreting the results. Responses from 482 senior leaders generated the names of 794 individuals and 587 organizations as sources of advice for improving resident care in long-term care facilities.

**Results:**

A single advice-seeking network appears to span the nation. Proximity exhibits a strong effect on network structure, with provincial inter-organizational networks having more connections and thus a denser structure than interpersonal networks. We found credible individuals and organizations within groups (opinion leaders and opinion-leading organizations) and individuals and organizations that function as weak ties across groups (boundary spanners and bridges) for all studied provinces and territories. A good deal of influence in the Canadian long-term care sector rests with professionals such as provincial health administrators not employed in long-term care facilities.

**Conclusions:**

The Canadian long-term care sector is tied together through informal advice-seeking relationships that have given rise to an emergent network structure. Knowledge of this structure and engagement with its opinion leaders and boundary spanners may provide a route for stimulating the adoption and effective implementation of best practices, improving resident care and strengthening the long-term care advice network. We conclude that informal relational pathways hold promise for helping to transform the Canadian long-term care sector.

## Background

The *diffusion of innovations* paradigm [1] suggests that the structure of relationships among the members of a social system, in combination with perceptions of innovations and the environmental context in which a social system is embedded, affects the decisions of members to adopt an innovation or not [2–9]. Especially when an innovation strikes potential adopters as important, they rely on credible others in their network of relationships for guidance before adoption or rejection [1, 10]. This fundamental importance of social influence in decision processes is well-established across academic disciplines and practical applications [1, 11, 12].

Having insight into the informal structure of an advice-seeking network is akin to having a key. Once in hand, the key can be used by an intervention team to help estimate where in a network interventions can best be seeded. An intervention team can also identify structural weaknesses in the network—such as a potential relationship that does not yet exist or two groups that might benefit from being tied together—that can be targeted for strengthening in an overall *designing for diffusion* framework [13]. The absence of a relationship can represent an opportunity for tying a network more closely together. In a complementary fashion, the spread of an innovation once it is introduced into a network can be accelerated by well-established relationships formed on the basis of co-location, a common background, employment in the same sector, or another basis for people to perceive similarity with each other [14]. This is especially the case for colleagues who are accessible and who we perceive to be trustworthy and/or an expert about a topic—informally influential opinion leaders [15]. In studies of knowledge diffusion, scholars have observed that central advice sources may act as opinion leaders, driving the spread of knowledge through the network [16, 17].

In this study, we sought to identify existing influence among directors of care in Canada’s long-term care (LTC; nursing home) sector and the extent to which informal advice-seeking relationships among them bind them as a network that spans the sector. An understanding of the extent to which this sector is informally interconnected could offer a new means for stimulating a national system transformation [18], which is our team’s distal goal. The sustainability of the LTC sector in Canada depends upon system transformation.

### The decentralized Canadian context

The formal structure of the Canadian LTC sector is complex and variable across provinces and territories. The LTC sector in Canada sits outside of the Canadian Medicare System, such that while many components of the health care system in Canada are publicly financed, the long-term care sector is financed through a combination of public and private contributions [19]. Provinces are responsible for how long-term care facilities organize, deliver, and monitor care. Their respective approaches have been shaped by where residential long-term care has been situated in the province’s evolution of health and social policy. Generally, provincial and territorial ministries have responsibility for legislation, regulations, standards, and policies. Presently, residential long-term care is situated in the “health” portfolio of provincial governments, with the exception of New Brunswick where it is under social development. In a few provinces, ministries own and operate long-term care facilities (e.g., Prince Edward Island) whereas in others, the owner-operator model may include the regional health authority (e.g., Saskatchewan), not-for-profit only (e.g., New Brunswick) or a mix of nonprofit and for-profit (e.g., Nova Scotia, Manitoba).

From staff and resident perspectives, differences exist across the country as well. Direct care staff vary in how they are identified, the education requirement for entry to practice, and the extent to which their workplace is unionized [20]. Entry to LTC is based on provincial/territorial criteria, supported by centralized and coordinated entry, and in many jurisdictions, a standardized assessment (the interRAI-Home Care^1^) is used as part of the LTC application process to inform eligibility and priority. All residents pay some portion of accommodation costs, often based on their income.

### Study rationale and purpose

In Canada, as in a number of countries, the proportion of people aged 65 and older is increasing, with projections suggesting a very substantial proportion of future populations in this age group. The number of Canadians 65 and older will more than double to 10.4 million by 2036 [21]. Already, Canadian LTC residents are increasingly older and more frail with multiple chronic conditions and specialized needs, and most have dementia [22].

The purpose of our project was to identify existing advice-seeking networks among LTC facilities that are within Canada’s residential LTC sector by using social network analysis. Our goal was to inform future efforts to disseminate transformative innovations using this knowledge. Our specific aims were to:

1. Identify the structure of existing informal inter-organizational and interpersonal relationships among 958 LTC facilities and LTC directors of care in Atlantic, Western, and Northern Canada.
2. Identify which LTC facilities and individuals within groups are most influential and which homes and individuals within the overall network link different groups together.

Our team has related aims, not dealt with here, of explaining why care improvement advice is sought (through a qualitative study) and studying the knowledge translation roles of our practitioner colleagues in system transformation. Our pan-Canadian team has worked together for several years within the Translating Research in Elder Care (TREC) program of research that has been explained in this journal [23–25] and that relies on a partnership model of applied research.

### Levels of analysis in social networks

Social network metrics allow for the analysis of nodes (single actors such as a person or an organization), how nodes are tied together, and analysis of networks as a whole. We conceptualized and wanted to compare an interpersonal network with an inter-organizational network because LTC leaders—like actors in other industries—may well look to both individuals and to organizations when they are considering the adoption of a care improvement innovation. We do this while acknowledging that in a LTC leader’s mind, the two are blended; he or she has one ego-centric network in mind consisting of comparative and aspirational sources [26, 27]. This reference group is likely composed of a care director’s set of known colleagues, along with some number of organizations that are watched and admired. Such organizations may be both other LTC facilities that are considered progressive or highly reputable and other types of organizations such as provincial health departments, quality assurance organizations, and university departments of geriatrics.

## Methods

### Study population

The study population consisted of one senior leader (most with the job title of Director of Care or Director of Nursing) from each of the 958 LTC facilities operating at the time of data collection in the Canadian provinces of Newfoundland and Labrador, Prince Edward Island, Nova Scotia, and New Brunswick in Atlantic Canada; British Columbia, Alberta, Saskatchewan, and Manitoba in Western Canada; and the territories of Yukon, Nunavut, and the Northwest Territories in Northern Canada. We defined “LTC facility” as a residential long-term care setting for older adults, commonly those aged 65 and older, that offers 24-h on-site personal care, nursing care, and housekeeping services. Our facility sample was a census one. Eligible LTC facilities were first identified using the *Guide to Canadian Healthcare Facilities* (2012) and then verified via consultation with regional LTC professionals. We chose the senior leaders because they had decision-making responsibility for clinical care and for implementing innovations that influence best practice use, evidence-based decision-making, and resident care quality.

### Data collection and measures

Data collection occurred between November 2014 and May 2015 via distribution of an online survey instrument and followed the Dillman method of tailored survey design [28]. The survey was available in English and French. To pilot test the survey instrument, we recruited four LTC leaders in Edmonton and six in Atlantic Canada to complete the survey and participate in cognitive debriefing, with one participant completing the survey in French. Feedback from the pilot testing resulted in refinement of survey format, instructions, and question wording.

We designed the survey to take 10 min to complete and included questions about advice-seeking behavior, demographics, and current employment and employment history. We assessed advice-seeking behavior at both the interpersonal and inter-organizational levels. For interpersonal advice seeking, we asked the participants to list individuals external to their LTC facility whose advice they seek or behavior they monitor about delivery of quality care, care improvement, and innovation. The respondents were instructed that these interpersonal sources of advice could include people who work in a LTC facility or those who work in another setting such as government, not-for-profit organizations, or industry. The participants could list up to three individuals (from most- to second-most- to third-most-valued source of advice) and were asked to specify the individuals’ job titles and organizational affiliations. For inter-organizational advice seeking, we asked the participants to list LTC facilities whose example or reputation they followed with respect to delivery of quality care, care improvement, and innovation. The participants could list up to three organizations in the order of most valued sources.

We collected employment data by asking the participants to indicate their primary organizational affiliation, if they had responsibility for more than one facility, how long they had worked in long-term care over their career, and how long they had worked in their position at their current LTC facility. We also asked the participants to specify whether their primary facility was free-standing or co-located with another health care facility and for the last three organizations in which they had worked. We asked for demographic information on job title, gender, age, highest level of education achieved, and professional background.

In addition to the data collected via survey instrument, we collected three variables describing the individuals and LTC facilities in the study population from publicly available records. The first variable was the health authority in which each individual worked or in which each LTC facility was located. We used the health authority data to examine whether patterns in advice-seeking relationships were influenced by geographic proximity. The second variable was the owner-operator model of each LTC facility in the study. Following the protocol of our parent research program, TREC, we classified owner-operator models in three categories: public not-for-profit, voluntary (e.g., faith based) not-for-profit, and private for-profit [23]. On the advice of regional experts, we used a fourth category, private not-for-profit, to characterize ownership of the majority of LTC facilities in one province, New Brunswick. The third variable was the size of each LTC facility, as measured by number of beds in each facility. Again, following the TREC protocol, we classified the size in three categories: small (fewer than 80 beds), medium (80–120 beds), and large (more than 120 beds).

A final variable for the interpersonal networks, *professional role*, was created using the job title and organizational affiliation information collected for each survey participant and each individual nominated as a source of advice.

### Analysis

We cleaned the collected survey data to remove duplicate responses and incomplete responses. Complete responses provided the respondent’s name, job title, primary LTC affiliation, and the nomination of at least one individual or organization (outside of the respondent’s own focal organization) as a source of advice. Survey respondents who reported working at more than one LTC facility were represented as one node only in the interpersonal network, but as multiple nodes (one node for each LTC facility at which the respondent worked) in the inter-organizational network. We then created adjacency matrices for the interpersonal and inter-organizational networks in each of the provinces and territories, in which “1” indicated that an advice or social modeling relationship existed between two senior leaders or two LTC facilities and “0” indicated the absence of a relationship. The matrices were constructed such that the ego in the dyad was the advice seeker (the survey respondent or the respondent’s primary LTC facility) and the alter was the advice source or model (an individual who the respondent identified as a valued source of advice or an organization that the respondent identified as a model of quality care). We then used the two adjacency matrices as data files in the network analysis.

We performed analyses at two levels: at the level of each province and territory and at the pan-Canadian or whole-network level. The employment and demographic data were analyzed by calculation of descriptive statistics, using SPSS version 23. The network data were analyzed by calculation of network descriptive statistics at the whole-network, province or territory, and nodal (individual and organizational) levels using SPSS, UCINET version 6, and Gephi version 0.9. We created network visualizations using Gephi and ArcGIS.

At the whole-network level, we measured the number of types of nodes and ties, density, and in-degree centralization. Network density is calculated by dividing the number of observed ties in the network by the number of possible ties that could exist, if all nodes were connected to all other nodes [29]. In many social networks, density is quite low [30] and when considered in isolation the measure is not particularly meaningful. It does, however, offer a useful measure to compare the relative connectedness of a number of different networks, as was our objective here with the pan-Canadian analysis.

At the nodal level, because of our interest in best practice diffusion, we sought to identify individuals and LTC facilities that play key roles in the flow of advice through the networks: opinion leaders and boundary spanners. We identified nodes in the interpersonal and inter-organizational networks as opinion leaders on the basis of their in-degree centrality scores. In-degree centrality is a simple count of the number of incoming ties, or relationships, a node receives [29]. It is the most commonly used measure of opinion leadership [31], but little formal consensus exists on the appropriate threshold, based on in-degree measures, for identifying the number of opinion leaders in a particular network [16, 32]. Accordingly, we tested the appropriateness of several different thresholds for our data and found that for each provincial and territorial network, an in-degree threshold of at least two standard deviations above the mean in-degree score of all nodes in the network offered the best fit for our data.

Boundary spanners are individuals or organizations that connect two or more groups in the larger network. Network and diffusion scholars have investigated the association between diffusion and the presence of boundary spanners who “span” structural holes between nodes or groups of nodes in the network [33]. Opinion leaders who occupy central positions, for example, sometimes act as boundary spanners by virtue of the greater numbers of others connected to them. They can locate relevant and diverse knowledge and then exchange it with others. Less central actors often also act as boundary spanners, as in a person peripherally connected to two different groups who acts as a link between the two. We identified boundary spanners in the interpersonal and inter-organizational networks using the betweenness centrality score of each node. Betweenness centrality assesses the degree to which a node lies on the shortest path connecting others in the network. To count the number of boundary spanners in each network, we applied the same formula that we used for counting opinion leaders: a betweenness centrality threshold of at least two standard deviations above the mean betweenness centrality score of all nodes in the network.

## Results

### Survey respondents

Because of response rates of less than 30% in Yukon Territory, Nunavut Territory, and the province of Newfoundland and Labrador, we excluded data from these areas from our analysis. From the 926 senior LTC leaders surveyed in the remaining eight provinces and territories, we collected a total of 482 complete responses for an overall response rate of 52%. Specific response rates for each province and territory included in the analysis are reported in Table 1 and ranged from 41 to 100%.

**Table 1:** Response rates and descriptive statistics for survey participants [*N* (%), except where noted]

	NS	PE	NB	MB	SK	AB	BC	NT	*M*	Total
*N* LTC facilities	88	16	65	128	156	175	290	8	115.75	926
Responses	53 (60)	12 (75)	48 (74)	83 (65)	68 (44)	90 (51)	120 (41)	8 (100)	60.25	482 (52)
Gender										
Women	44 (83)	9 (75)	40 (83)	65 (78)	58 (85)	70 (78)	96 (80)	4 (50)	48.25	386 (80)
Men	7 (13)	2 (17)	3 (6)	10 (12)	5 (7)	10 (11)	14 (12)	2 (25)	6.63	53 (11)
Missing^a^	2 (4)	1 (8)	5 (10)	8 (10)	5 (7)	10 (11)	10 (8)	2 (25)	5.38	43 (9)
Age										
20–39	3 (6)	2 (17)	5 (10)	6 (7)	4 (6)	8 (9)	11 (9)	1 (13)	5.00	40 (8)
40–59	42 (79)	8 (67)	34 (71)	65 (78)	50 (74)	54 (60)	84 (70)	4 (50)	42.63	341 (71)
60+	7 (13)	1 (8)	7 (15)	8 (10)	10 (15)	20 (22)	17 (14)	1 (13)	8.88	71 (15)
Missing	1 (2)	1 (8)	2 (4)	4 (5)	4 (6)	8 (9)	8 (7)	2 (25)	3.75	30 (6)
Education										
Diploma/certificate	23 (43)	3 (25)	4 (8)	35 (42)	30 (44)	37 (41)	41 (34)	3 (38)	22.00	176 (37)
Bachelors	26 (49)	8 (67)	37 (77)	35 (42)	26 (38)	30 (33)	36 (30)	1 (13)	24.88	199 (41)
Graduate	3 (6)	0	5 (10)	9 (11)	5 (7)	15 (17)	33 (28)	2 (25)	9.00	72 (15)
Missing	1 (2)	1 (8)	2 (4)	4 (5)	7 (10)	8 (9)	10 (8)	2 (25)	4.38	35 (7)
Professional background										
Nursing	51 (96)	11 (92)	47 (98)	64 (77)	48 (71)	69 (77)	87 (73)	4 (50)	47.63	381 (79)
Business	1 (2)	0	0	9 (11)	10 (15)	5 (6)	12 (10)	0	4.63	37 (8)
Other	0	0	0	6 (7)	6 (9)	8 (9)	13 (11)	2 (25)	4.38	35 (7)
Missing	1 (2)	1 (8)	1 (2)	4 (5)	4 (6)	8 (9)	8 (7)	2 (25)	3.63	29 (6)
Works at >1 facility										
No	50 (94)	9 (75)	47 (98)	67 (81)	57 (84)	79 (88)	102 (85)	7 (88)	52.25	418 (87)
Yes	3 (6)	3 (25)	1 (2)	16 (19)	11 (16)	11 (12)	18 (15)	1 (13)	8.00	64 (13)
Missing	0	0	0	0	0	0	0	0	0	0
Facility management^b^										
Stand-alone	45 (85)	9 (75)	45 (94)	61 (73)	50 (74)	65 (72)	89 (74)	3 (38)	45.88	367 (76)
Co-located	8 (15)	3 (25)	3 (6)	21 (25)	18 (27)	25 (28)	31 (26)	5 (63)	14.25	114 (24)
Missing	0	0	0	1 (1)	0	0	0	0	0.13	1 (0)
Years worked [*M (SD)*]										
In LTC	14.59 (9.80)	16.23 (10.05)	15.34 (9.24)	16.93 (11.47)	16.52 (9.43)	15.18 (10.63)	14.86 (9.61)	9.33 (11.15)	14.87	−
Missing	1 (2)	1 (8)	1 (2)	6 (7)	4 (6)	8 (9)	14 (12)	2 (25)	4.63	37 (8)
In current job	6.75 (7.28)	6.32 (5.88)	7.68 (4.95)	4.87 (4.19)	5.58 (6.31)	6.23 (11.77)	4.14 (3.38)	3.30 (3.52)	5.61	−
Missing	1 (2)	1 (8)	1 (2)	7 (8)	4 (6)	12 (13)	11 (9)	2 (25)	4.88	39 (8)

*NS* Nova Scotia, *PE* Prince Edward Island, *NB* New Brunswick, *MB* Manitoba, *SK* Saskatchewan, *AB* Alberta, *BC* British Columbia, *NT* Northwest Territories, *LTC* long-term care

^a^The percentage of missing data for each variable was calculated by using as the denominator the total number of responses received in a particular geographic area. For example, the percentage of missing data for the gender variable in Nova Scotia was 2/53 = 4%

^b^Refers to management model of participant’s primary facility. “Stand-alone” refers to a free-standing facility that has its own management staff, whereas “co-located” refers to a facility that shares management staff and resources with another, typically non-LTC, facility

The complete demographic and employment characteristics of the survey respondents are also summarized in Table 1. A majority of the senior LTC leaders who responded to the survey were women aged 40 to 59 (71%) with a professional background in nursing (79%). Their mean years worked in the long-term care sector was about 15 years, and mean years worked in their current position was about 6 years.

### Network characteristics and measures

**Fig. 1:**
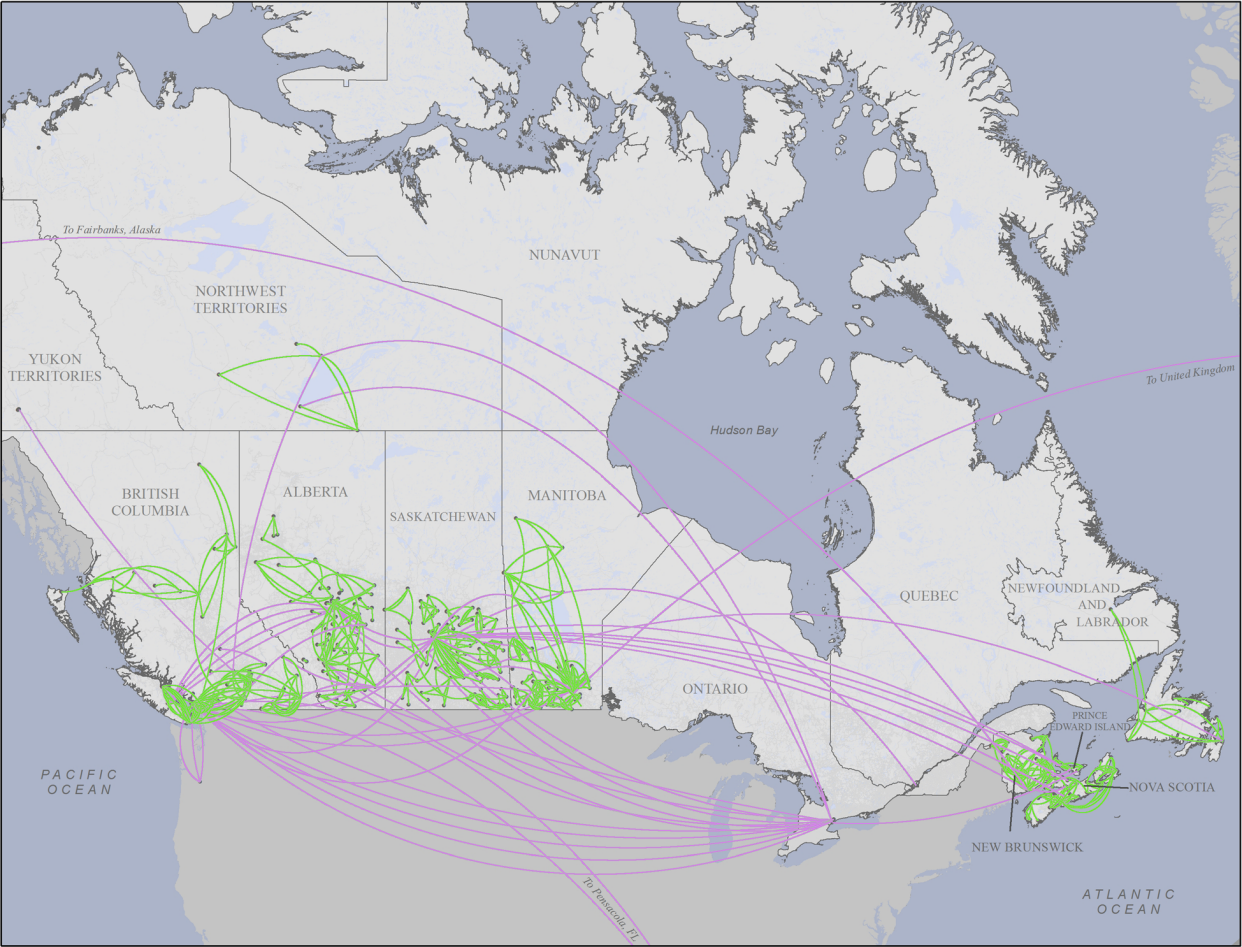
Pan-Canadian inter-organizational network. The *black circles* represent LTC facilities, and the *green* and *purple lines* represent advice relationships between them. The *green lines* indicate *intra*-provincial or territorial relationships, and the *purple lines* indicate *inter*-provincial or territorial relationships. Note that Ontario and Quebec were not included in the study sample

Responses from the 482 senior LTC leaders generated the names of 794 individuals and 587 organizations as sources of advice and example for social network analysis. Figure 1 presents a visualization of the inter-organizational advice network across Canada, illustrating the geographic scope of the study and the spatial distribution of advice seeking. Advice relationships extend across provinces and territories to create a single national inter-organizational network, but these inter-provincial relationships are relatively rare compared with intra-provincial relationships and account for only 5% of links in the network. Most of the social influence for care improvement in the long-term care sector appears to occur intra-provincially and locally. This general geographic pattern of advice relationships in the inter-organizational network also applies to the interpersonal network.

Tables 2 and 3 present measures describing the structures of the interpersonal and inter-organizational advice networks, respectively. The interpersonal advice network is composed of 1140 individuals, ranging from 19 in the Northwest Territories to 300 in British Columbia. The network has 1181 links, with just a small fraction (3%) of these crossing provincial or territorial boundaries; this reinforces the geographic pattern of advice seeking observed in the visualization of the inter-organizational network displayed in Fig. 1. Interpersonal network density across all provinces and territories is low, with the highest densities in the areas with the smaller populations, as is often observed in social networks.

**Table 2:** Measures for interpersonal advice network, by province and territory

	NS	PE	NB	MB	SK	AB	BC	NT	*M* (SD)	Total
Network Level										
*N* nodes	135	32	93	181	155	225	300	19	142.50 (95.03)	1140
*N* advice sources	101	25	73	116	103	153	211	12	99.25 (64.91)	794
*N* advice seekers	50	13	47	77	64	88	116	7	57.75 (36.78)	462
*N* ties	134	36	124	195	166	214	296	16	147.63 (92.22)	1181
*N* inter-provincial ties	0	1	2	2	4	11	7	3	3.75 (3.62)	30
Density	0.007	0.036	0.014	0.006	0.007	0.004	0.003	0.047	0.016 (0.017)	
In-degree centralization	0.08	0.13	0.07	0.06	0.05	0.02	0.02	0.19	0.08 (0.06)	
Nodal level										
In-degree centrality										
*N* opinion leaders^a^	2	1	5	6	12	14	9	1	6.25 (5.01)	50
In-degree, all nodes [*M* (SD)]	0.99 (1.18)	1.13 (1.01)	1.33 (1.42)	1.07 (1.61)	1.07 (1.32)	0.95 (1.01)	0.99 (1.03)	0.84 (0.96)	1.05 (0.14)	
In-degree, opinion leaders [*M* (SD)]	8.00 (5.66)	5.00 (−)	6.00 (1.23)	8.33 (1.63)	4.75 (1.29)	4.00 (1.04)	5.00 (1.00)	4.00 (−)	5.64 (1.69)	
Betweenness centrality										
*N* boundary spanners^b^	6	2	6	13	4	9	10	1	6.38 (4.10)	51
Betweenness centrality, all nodes [*M* (SD)]	0.50 (1.88)	1.28 (3.00)	7.05 (21.69)	0.24 (0.85)	0.27 (1.35)	0.29 (1.27)	0.42 (1.83)	0.11 (0.46)	1.27 (2.36)	
Betweenness centrality, boundary spanners [*M* (SD)]	8.50 (2.59)	10.50 (0.71)	84.00 (22.65)	2.96 (1.22)	6.50 (5.74)	5.67 (2.83)	8.95 (4.34)	2.00 (−)	16.14 (27.58)	

*NS* Nova Scotia, *PE* Prince Edward Island, *NB* New Brunswick, *MB* Manitoba, *SK* Saskatchewan, *AB* Alberta, *BC* British Columbia, *NT* Northwest Territories

^a^Opinion leaders were defined as all nodes with in-degree centrality scores of at least two standard deviations above the mean

^b^Boundary spanners were defined as all nodes with betweenness centrality scores of at least two standard deviations above the mean

**Table 3:** Measures for inter-organizational advice network, by province and territory

	NS	PE	NB	MB	SK	AB	BC	NT	*M (SD)*	Total
Network Level										
*N* nodes	80	20	59	133	119	151	217	13	99.00 (69.47)	792
*N* advice sources	66	16	52	99	85	103	158	8	73.38 (49.15)	587
*N* advice seekers	48	14	49	90	69	78	119	8	59.38 (37.55)	475
*N* ties	129	36	139	240	187	181	303	15	153.75 (96.74)	1230
*N* inter-provincial ties	3	4	2	8	11	11	15	7	7.63 (4.53)	61
Density	0.020	0.095	0.041	0.014	0.013	0.008	0.006	0.096	0.037 (0.038)	
In-degree centralization	0.07	0.18	0.13	0.10	0.11	0.03	0.03	0.26	0.11 (0.08)	
Nodal level										
In-degree centrality										
*N* opinion leaders^a^	4	1	4	6	3	11	9	1	4.88 (3.60)	39
In-degree, all nodes *[M (SD)]*	1.61 (1.44)	1.80 (1.47)	2.36 (2.20)	1.81 (1.99)	1.60 (1.92)	1.20 (1.25)	1.40 (1.37)	1.15 (1.28)	1.62 (0.39)	
In-degree, opinion leaders *[M (SD)]*	5.75 (0.96)	5.00 (−)	8.75 (0.96)	8.17 (3.06)	10.33 (4.04)	4.36 (0.50)	5.56 (0.53)	4.00 (−)	6.49 (2.30)	
Betweenness centrality										
*N* boundary spanners^b^	7	2	3	9	5	6	16	2	6.25 (4.65)	50
Betweenness centrality, all nodes [*M* (SD)]	27.09 (57.38)	7.90 (11.88)	37.53 (67.65)	12.38 (26.45)	4.99 (16.50)	7.62 (22.76)	4.54 (10.40)	0.85 (2.08)	12.86 (12.75)	
Betweenness centrality, boundary spanners [*M* (SD)]	185.38 (32.01)	36.25 (3.18)	274.44 (61.88)	94.94 (25.01)	67.00 (43.27)	101.25 (43.06)	35.53 (10.61)	5.50 (0.71)	100.04 (89.43)	

*NS* Nova Scotia, *PE* Prince Edward Island, *NB* New Brunswick, *MB* Manitoba, *SK* Saskatchewan, *AB* Alberta, *BC* British Columbia, *NT* Northwest Territories

^a^Opinion leaders were defined as all nodes with in-degree centrality scores of at least two standard deviations above the mean

^b^Boundary spanners were defined as all nodes with betweenness centrality scores of at least two standard deviations above the mean

We identified 50 opinion leaders in the interpersonal advice network, with the count in provinces and territories ranging from 1 to 14. In-degree centrality scores averaged about 1 for all individuals and about 6 for opinion leaders. Network centralization was highest in the Atlantic provinces and in the Northwest Territories and lowest in the Western provinces. We also identified 51 boundary spanners in the interpersonal network, with a count across the provinces and territories ranging from 1 to 13. The average betweenness centrality score was 1 for all individuals and 16 for boundary spanners.

**Fig. 2:**
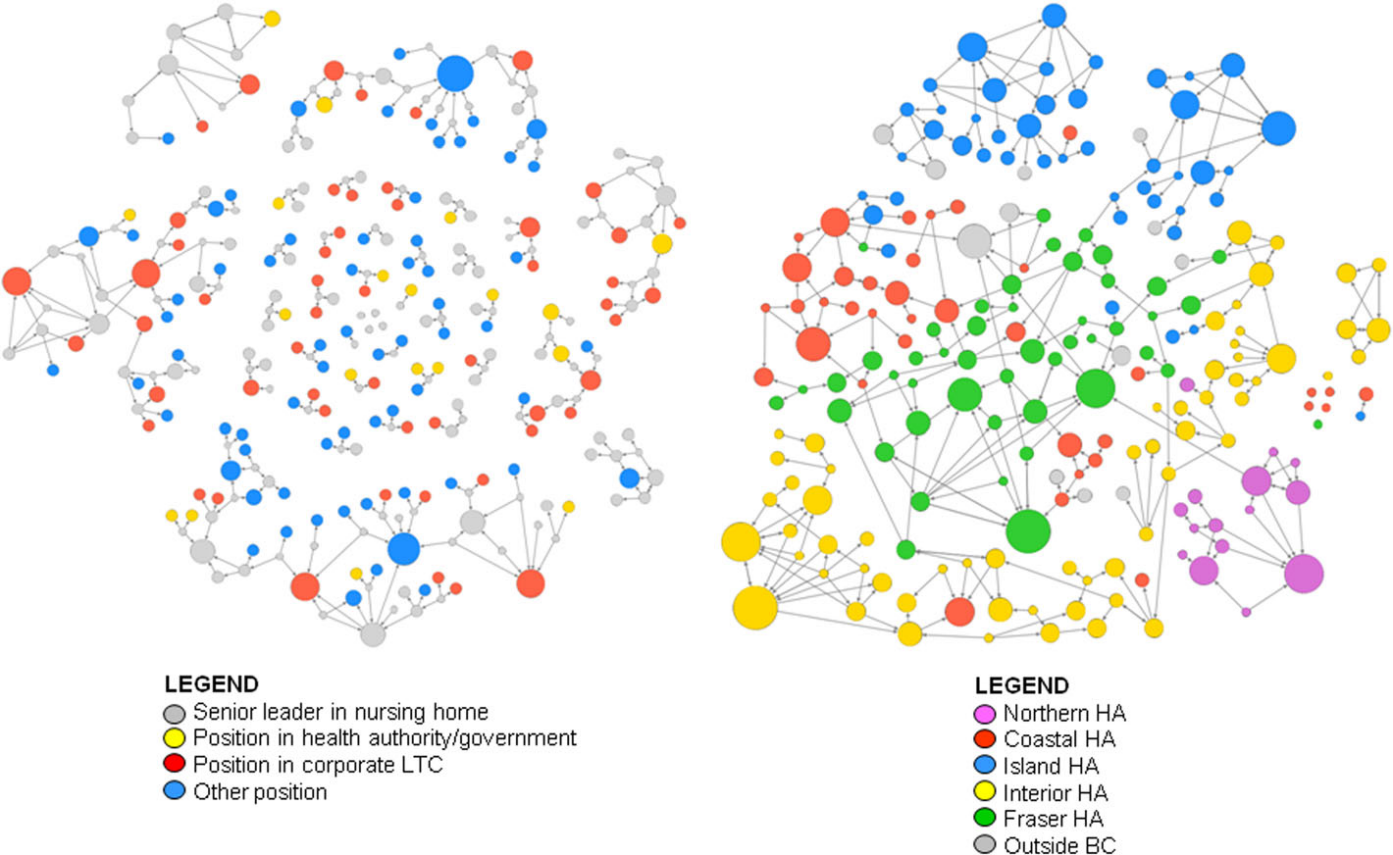
Interpersonal and inter-organizational networks in British Columbia. The interpersonal network (*left*) is color-coded by an individual’s organizational affiliation, and the inter-organizational network (*right*) is color-coded by LTC facility geographic location. Nodes are sized according to in-degree centrality score, such that larger nodes have higher in-degree scores and the largest nodes represent opinion leaders. *LTC* long-term care, *HA* health authority, *BC* British Columbia

Descriptive analysis of the professional role data collected for each individual in the interpersonal network is reported in Table 4. As this table indicates, a substantial proportion of individuals in the provinces and territories have titles other than LTC senior leader or director of care. Many of the individuals nominated as sources of advice, in fact, were those working in corporate LTC positions, in regional health authorities and provincial governments, and in consultant or expert roles in the LTC sector. The British Columbia interpersonal network visualized in the left panel of Fig. 2 illustrates this finding, with nodes in the network color-coded according to professional role.

**Table 4:** Professional roles for individuals in interpersonal advice network [*N* (%), except where noted]

	NS	PE	NB	MB	SK	AB	BC	NT	*M*	Total
*N* individuals in network	135	32	93	181	155	225	300	19	142.50	1140
Senior leadership position in an LTC facility (e.g., director of care)^a^	81 (60)	18 (56)	58 (62)	113 (62)	90 (58)	125 (56)	160 (53)	7 (37)	81.50	652 (57)
Position in corporate level of an organization providing LTC	10 (7)	2 (6)	3 (3)	9 (5)	1 (1)	29 (13)	19 (6)	0	9.13	73 (6)
Chief executive officer/president/vice president	2	2	2	1	0	8	3	0	2.25	18
Quality improvement/clinical services	3	0	1	2	0	9	3	0	2.25	18
General director/regional leader	5	0	0	6	1	12	13	0	4.63	37
Position in regional health authority or government	12 (9)	7 (22)	5 (5)	34 (19)	43 (28)	43 (19)	54 (18)	5 (26)	25.38	203 (18)
Director, seniors health/continuing care	1	1	0	10	13	31	19	2	9.63	77
Director, education/best practice/ quality improvement	0	2	0	6	3	12	5	1	3.63	29
Manager, case coordination/care coordination/access	5	2	5	2	2	0	10	0	3.25	26
Licensing and review	1	0	0	3	0	0	9	0	1.63	13
Other	5	2	0	13	25	0	11	2	7.25	58
Other position/affiliation	32 (24)	5 (16)	27 (29)	25 (14)	21 (14)	28 (12)	67 (22)	7 (37)	26.50	212 (19)
Therapist, physical/occupational/ recreational	0	0	3	1	0	3	3	1	1.38	11
Mental health clinician, therapist/behavioral	6	0	2	1	0	3	6	2	2.50	20
Educator, best practice/clinical practice	3	0	1	4	4	2	9	0	2.88	23
Specialist, wound care/infection control	1	0	1	3	2	3	10	0	2.50	20
Clinician, physician/pharmacist/nurse	15	2	8	5	4	5	13	2	6.75	54
Other	7	3	12	11	11	12	26	2	10.50	84

*NS* Nova Scotia, *PE* Prince Edward Island, *NB* New Brunswick, *MB* Manitoba, *SK* Saskatchewan, *AB* Alberta, *BC* British Columbia, *NT* Northwest Territories, *LTC* long-term care

^a^Percentages are provided for the four main categories of professional roles only, and not for the specific job titles

Table 5 reports on the ownership and size of LTC facilities in the inter-organizational advice network. Public not-for-profit was the most common owner-operator model (43%), then voluntary not-for-profit (24%), and private for-profit (22%). About half of the LTC facilities in the network were small, with fewer than 80 beds, and the other half was split equally between medium and large facilities. Inspection of sociograms by province and territory, coded according to ownership and size, suggested no clear influence of these variables on network structure.

**Table 5:** Owner-operator model and number of beds for LTC facilities in inter-organizational advice network [*N* (%)]

	NS	PE	NB	MB	SK	AB	BC	NT	*M*	Total
*N* LTC facilities in network	80	20	59	133	119	151	217	13	99.00	792
Owner-operator										
Public not-for-profit	11 (14)	8 (40)	1 (2)	73 (55)	87 (73)	69 (46)	82 (38)	10 (77)	42.63	341 (43)
Private for-profit	38 (48)	8 (40)	0	17 (13)	4 (3)	46 (30)	60 (28)	0	21.63	173 (22)
Voluntary not-for-profit	30 (38)	2 (10)	1 (2)	40 (30)	24 (20)	32 (21)	61 (28)	0	23.75	190 (24)
Private not-for-profit^a^	NA	NA	55 (93)	NA	NA	NA	NA	NA	6.88	55 (7)
Missing^b^	1 (1)	2 (10)	2 (3)	3 (2)	4 (3)	4 (3)	14 (6)	3 (23)	4.13	33 (4)
No. Beds										
0–79	44 (55)	9 (45)	41 (69)	74 (56)	87 (73)	73 (48)	76 (35)	7 (54)	51.38	411 (52)
80–120	15 (19)	1 (5)	5 (8)	24 (18)	13 (11)	21 (14)	60 (28)	0	17.38	139 (18)
>120	14 (18)	0	11 (19)	25 (19)	10 (8)	35 (23)	47 (22)	0	17.75	142 (18)
Missing	7 (9)	10 (50)	2 (3)	10 (8)	9 (8)	22 (15)	34 (16)	6 (46)	12.50	100 (13)

*NS* Nova Scotia, *PE* Prince Edward Island, *NB* New Brunswick, *MB* Manitoba, *SK* Saskatchewan, *AB* Alberta, *BC* British Columbia, *NT* Northwest Territories, *LTC* long-term care

^a^Applicable to New Brunswick only

^b^The percentage of missing data for each variable was calculated by using as the denominator the total number of LTC facilities in a particular geographic area (i.e., the first number in the column). For example, the percentage of missing data for the owner-operator variable in Nova Scotia was 1/80 = 1%

In comparison to the interpersonal advice network, the inter-organizational network had fewer nodes—the respondents named fewer distinct organizations than individuals. Given that the number of possible individuals to name is larger than the number of possible organizations, this is unsurprising. In the interpersonal network, 1140 individuals with 1181 links clustered into 87 groups; in the inter-organizational network, 792 organizations with 1230 links clustered into 19 groups. In each province and territory, network density scores were higher in the inter-organizational network than in the interpersonal network. This difference between the two networks is illustrated perhaps most dramatically by the data from British Columbia. Figure 2 depicts a side-by-side comparison of sociograms for the British Columbia interpersonal and inter-organizational networks, illustrating that the inter-organizational network appears much more dense and interconnected than the interpersonal network. Quantitatively, the density score was 0.003 for the interpersonal network (Table 2) and 0.020 for the inter-organizational network (Table 3).

The inter-organizational advice network is similar to the interpersonal network in highlighting clear opinion-leading organizations and boundary spanning organizations. We identified 39 opinion-leading organizations, with in-degree centrality scores averaging about 2 for all organizations and 6 for opinion-leading organizations. We also identified 50 boundary spanning organizations, with an average betweenness centrality score of 13 for all organizations and 100 for boundary spanning organizations.

A second similarity between the interpersonal and inter-organizational networks emerged in analysis of the data on health authority geography in each province and territory. At the province and territory level, inspection of sociograms color-coded by health authority geography suggested that opinion-seeking individuals and organizations looked to others who are geographically proximate to them and within their same health authority. In Fig. 2, the sociogram of the British Columbia inter-organizational network in the right panel offers an example of the extent to which senior leaders look within their own health authority for models of care improvement. This result not only offers an important insight for designing best practice dissemination initiatives in Canadian long-term care but is also not surprising. Geographic proximity often plays an important role in the structuring of advice and other social networks in numerous contexts.

### Limitations

These data embed some limitations. Two of Canada’s provinces (Ontario and Quebec) are not represented, response rate was partial, and data collection was cross-sectional. While partial response rate to a large voluntary survey can always be expected, partial response rates are cause for caution in interpreting social network analyses. Ontario and Quebec, the provinces not yet represented in our data collection, are populous with many LTC facilities, so our structural understanding of advice seeking about LTC care improvement currently has this important limitation. We hope to address this deficiency in future waves of data collection. This would also enrich our cross-sectional first take at illuminating the structure of this relational network and how it may change as followers identify new opinion leaders; as individuals retire, relocate, and take on new jobs; and as organizations come and go.

As with any data collection procedure, certain aspects of our data are by-products of instrumentation. For example, the respondents were asked to name three individuals whose advice they most value and three LTC facilities whose example they follow, as the basis for social network analysis. In consequence, many four-node groups appear, and many nodes appear with three ties to others. Merely changing the instruction (to two or four) would have altered the results but not, we believe, in fundamental ways; “top of the mind” nominations would likely stay the same.

Social network data such as we have presented here show relationships among people and among organizations as reported by the survey respondents. In sociograms, the eye is often drawn to those nodes with many ties (high in-degree scores for opinion leaders). What is less obvious are relationships that do not exist in the data because they were not reported by the respondents. Absence of a tie between two nodes may result from lack of a relationship or from non-report of an existing relationship. Because response rate was partial and because respondents could only report up to three individuals and three organizations, it is possible that many nodes not linked to each other in our data are in fact tied and that groups exhibiting “structural holes” between them actually are tied. While the relational strata we do see in our data are arguably the most important, because the respondents were instructed to list those others whom they considered most valued, ours is possibly a considerable under-reporting of the actual advice-seeking network for LTC improvement in Canada. This possibility is particularly so for those advice sources who are not employed in LTC facilities but rather work as provincial administrators, health system directors, and quality assurance experts and in other non-LTC positions. Because these types of key individuals were not in our sampling frame, we have little systematic information about them. Anecdotally, however, we and our knowledge translation partners know or know of these individuals. Through their own information-sharing and advice-seeking behaviors, these authority figures may function to tie Canada’s LTC sector together more strongly than Fig. 1 shows, at a cross-provincial supra-level that we cannot detect in the present data with our sampling frame.

## Discussion

Transformative system change is necessary in Canada’s LTC sector, given the aging population, health trends of those individuals, and the resultant implications for health care costs. We believe that if informal opinion leaders work with formal sector leadership in considering best practice adoption and implementation, the care provided in Canadian LTC facilities can be transformed more rapidly. Accordingly, in this study, we collected sociometric (“who-to-whom”) data from directors of care in Canadian LTC facilities in 11 of Canada’s 13 provinces and territories. Our objective was to describe the extent and structure of advice-seeking networks among these facility directors. Our longer range intent is to combine these data about advice-seeking networks with knowledge translation strategies to accelerate the adoption of effective practices across Canada’s LTC facilities.

Our results suggest two main themes. *First*, physical proximity matters in LTC care improvement advice-seeking. Directors of care seek advice about care improvement from those who are nearby, both in terms of being employed in the same city and region and in terms of working under the jurisdiction of the same health authority. A second, possible proximity effect may manifest in terms of LTC facility ownership, but this is less clear in our data than is grouping by co-location and by health authority. Our results suggest that even in the age of social media and ready online information, care professionals still look to credible others who tend to be physically nearby. Of course, even those whose are physically proximate routinely communicate through text messaging, voice calls, Facebook posts, and email, enjoying an electronic form of proximity in which accessibility occurs through social media [34].

A *second* theme in our results is that directors of care in LTC facilities learn about ways to improve care both from conversation with and social modeling by individuals and from monitoring what other organizations are doing and advocating. With this blended individual and organizational reference group, directors of care can float the idea of adopting a new practice in their LTC facilities, comparing how care is pursued in their facilities with the aspirational standard of reference group facilities and recommendations. They do this through talk and messaging and observation, as well as through looking to see how the new practice is being received by other organizations. Are they trying it? Do they think it is a good idea? The use of reference groups by individuals for help in deciding whether to try a new practice is a reason why we asked the respondents for several interpersonal advice sources and several organizational advice sources. Taken together, any one director of care’s answers gives us a glimpse of their reference group for issues of care improvement.

Our separation of interpersonal sources of advice from inter-organizational sources of advice highlights structural differences between them and variance across provinces. In general, we see higher degrees of integration (density) in our inter-organizational sociograms than we do with our interpersonal sociograms. Figure 2 gives an example of this general pattern that we see in our data for other provinces, too. At provincial levels, inter-organizational ties appear stronger than interpersonal ties, with fewer small groups of two, three, or four nodes that appear unconnected to larger advice-seeking structures. This makes sense; even if a director of care does not know a particular person at another LTC facility, she can still look to that facility as a source of ideas. The converse is very unlikely because directors of care will almost always know the organization to which an individual belongs. We believe that this is a novel operationalization of social influence and one that in this case produces findings that have high utility for decision-makers in the health system. Knowing which individuals and which organizations collectively are best positioned to help in a dissemination and change effort is quite advantageous. What we do not know from the present analysis is the strength of belief or credibility that advice seekers vest in individuals versus organization. This is an aim of our tandem qualitative study. Knowing a friend and colleague might be expected to outweigh the influence of knowing what an organization is doing because social exchanges carry unspecified obligations to one another [35] that can accumulate into strong trusting relationships [36].

We are working with our knowledge translation colleagues in Canada’s provinces and territories to further interpret these results with the benefit of professional insight. We are also discussing ways in which LTC leaders may find unique value in data such as these for their own purposes in training, continuing education, and strategic decision-making.

## Conclusions

Our results suggest that a single advice-seeking network on the topic of improving resident care in long-term care (nursing home) facilities spans the nation of Canada. Advice-seeking relationships are relatively strong within province and weaker between province, with identifiable opinion leaders and boundary spanners. Proximity exhibits a strong effect on network structure, with provincial inter-organizational networks having more connections and thus a denser structure than interpersonal networks. We found credible individuals and organizations within groups (opinion leaders and opinion-leading organizations) and individuals and organizations that function as weak ties across groups (boundary spanners and bridges) for all studied provinces and territories. Considerable influence in the Canadian long-term care sector rests with professionals such as provincial health administrators not employed in long-term care facilities. Taken together, these are nontrivial and actionable results for our goal of working collaboratively with Canadian long-term care leaders to improve the state of practice in this critical sector.

## Abbreviations

LTC: Long-term care
RAI: Resident Assessment Instrument
TREC: Translating Research in Elder Care

## Footnotes

1
http://www.interrai.org/home-care.html
